# Small chloroplast-targeted DnaJ proteins are involved in optimization of photosynthetic reactions in *Arabidopsis thaliana*

**DOI:** 10.1186/1471-2229-10-43

**Published:** 2010-03-07

**Authors:** Kun-Ming Chen, Maija Holmström, Wuttinun Raksajit, Marjaana Suorsa, Mirva Piippo, Eva-Mari Aro

**Affiliations:** 1 Department of Biochemistry and Food Chemistry, Plant Physiology and Molecular Biology, University of Turku, FI-20014 Turku, Finland; 2 Institute of Crop Sciences, College of Agriculture and Biotechnology, Zhejiang University, 310029 Hangzhou, China

## Abstract

**Background:**

DnaJ proteins participate in many metabolic pathways through dynamic interactions with various components of these processes. The role of three small chloroplast-targeted DnaJ proteins, AtJ8 (At1 g80920), AtJ11 (At4 g36040) and AtJ20 (At4 g13830), was investigated here using knock-out mutants of *Arabidopsis thaliana*. Photochemical efficiency, capacity of CO_2 _assimilation, stabilization of Photosystem (PS) II dimers and supercomplexes under high light illumination, energy distribution between PSI and PSII and phosphorylation of PSII-LHCII proteins, global gene expression profiles and oxidative stress responses of these DnaJ mutants were analyzed.

**Results:**

Knockout of one of these proteins caused a series of events including a decrease in photosynthetic efficiency, destabilization of PSII complexes and loss of control for balancing the redox reactions in chloroplasts. Data obtained with DNA microarray analysis demonstrated that the lack of one of these DnaJ proteins triggers a global stress response and therefore confers the plants greater tolerance to oxidative stress induced by high light or methyl viologen treatments. Expression of a set of genes encoding enzymes that detoxify reactive oxygen species (ROS) as well as a number of stress-related transcription factors behaved in the mutants at growth light similarly to that when wild-type (WT) plants were transferred to high light. Also a set of genes related to redox regulation were upregulated in the mutants. On the other hand, although the three DnaJ proteins reside in chloroplasts, the expression of most genes encoding thylakoid membrane proteins was not changed in the mutants.

**Conclusion:**

It is proposed that the tolerance of the DnaJ protein knockout plants to oxidative stress occurs at the expense of the flexibility of photosynthetic reactions. Despite the fact that the effects of the individual protein knockout on the response of plants to high light treatment are quite similar, it is conceivable that both specific- and cross-talk functions exist between the three small chloroplast-targeted DnaJ proteins, AtJ8, AtJ11 and AtJ20.

## Background

Molecular chaperones participate in many important metabolic and survival reactions through dynamic interactions with various components of given processes. DnaJ proteins, also called J-domain proteins, function as molecular co-chaperones of Hsp70 and play an important role in protein folding, unfolding, and assembly under both normal and stress conditions as well as in cellular secretory pathways [1,2]. They are divided into three categories according to their domain composition [3] and have been identified in a variety of cellular compartments including cytosol [4], mitochondria [5], endoplastic reticulum [6], and chloroplasts [7]. Some of the DnaJ proteins also bind to the plasma membrane [8].

DnaJ proteins belong to a large family with several members: 22 in yeast [1], 41 in humans [9] and at least 89 in *Arabidopsis *[10]. According to our database searches at least 26 DnaJ proteins of *Arabidopsis *are predicted to have a chloroplast targeting signal and only few of them have been characterized. Based on only a few published studies it seems that the chloroplast-targeted DnaJ proteins participate in protein folding, unfolding and assembly processes [11]. Vitha *et al*. reported that ARC6, a chloroplast-targeted DnaJ-like protein localized to the plastid envelope membrane, participates in division of plastids probably by functioning in the assembly and/or stabilization of the plastid-dividing FtsZ ring in *Arabidopsis *[12]. It has been found that ATJ11, a chloroplast stroma localized DnaJ protein, is ubiquitously expressed in all plant organs examined so far [7]. DnaJ proteins found in the *Arabidopsis *chloroplast thylakoid proteome are likely to be important in thylakoid biogenesis [13]. Indeed, in *Chlamydomonas*, one chloroplast-targeted DnaJ protein was demonstrated to function in biogenesis of the thylakoid membrane [14].

Three DnaJ proteins, namely At1 g80920, At4 g36040 and At4 g13830, or AtJ8, AtJ11 and AtJ20, are small chloroplast-targeted DnaJ proteins in *Arabidopsis *with predicted molecular masses of 18.3-, 17.8- and 23.4-kD, respectively. These three proteins belong to the simplest group of the DnaJ proteins (type III) characterised by only one specific domain, the J-domain [1]. According to public microarray databases their gene expression patterns resemble each other [15]. We previously found that *AtJ8 *gene is upregulated in darkness [16] similar to that of *AtJ20 *gene (Supplementary material in [16]). To get more insights into the function of these small DnaJ proteins, the T-DNA insertion knockout mutants for AtJ8, AtJ11 and AtJ20 proteins, hereafter referred to as *j8*, *j11 *and *j20*, respectively, were isolated and characterised. The results provide evidence that the AtJ8, AtJ11 and AtJ20 proteins participate in optimization of various reactions of photosynthesis, and conversely, their absence triggers a global stress response.

## Results

### Characterization of DnaJ single mutants

**Figure 1:**
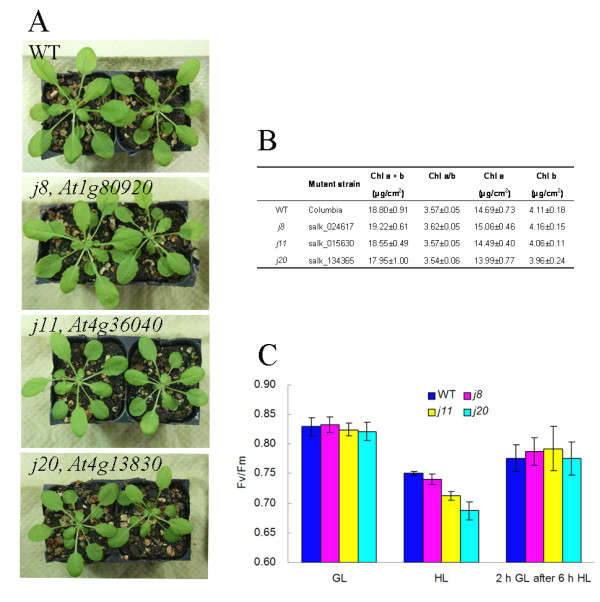
**Phenotypes of DnaJ protein knockout mutants**. A, Images of 4-week old wild-type (WT) and *j8*, *j11 *and *j20 *mutants; B, Contents of leaf chlorophyll in WT and the DnaJ mutants under growth light condition (120 μmol photons m^-2 ^s^-1^), the values are means ± SD (n = 10) of ten independent experiments; C, PSII photochemical efficiency of DnaJ mutants, the values are means ± SD (n = 10) of ten independent experiments. WT, wild-type; GL, growth light (120 μmol photons m^-2 ^s^-1^); HL, high light (1000 μmol photons m^-2 ^s^-1^).

*Arabidopsis *plants lacking a DnaJ protein AtJ8 (At1 g80920), AtJ11 (At4 g36040) or AtJ20 (At4 g13830) did not exhibit significantly different phenotypes compared to wild-type (WT) except for slightly stunted growth of the *j11 *and *j20 *mutants (Figure 1A and 1B). Photochemical efficiency of photosystem II (PSII) (Fv/Fm ratio) was not different between the WT and the DnaJ mutants under growth light (GL) conditions, whereas, it decreased somewhat more drastically in the mutants after exposure of 6 h to high light (HL) (1000 μmol photons m^-2 ^s^-1^), especially in *j11 *and *j20 *as compared to that in WT (Figure 1C). When plants were returned to GL conditions, the PSII photochemical efficiency recovered quickly and no differences were found between the WT and mutants (Figure 1C). The other mutant lines for the AtJ11 and AtJ20 proteins exhibited similar phenotypes as described above (Additional file 1).

### Localization of the three DnaJ proteins

**Figure 2:**
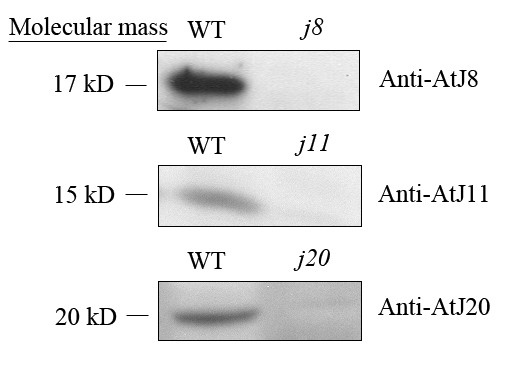
**Immunodetection of the three DnaJ proteins AtJ8, AtJ11 and AtJ20 in chloroplasts**. Chloroplasts were isolated from the leaves of WT and respective mutants after 3 h treatment in darkness. Total chloroplast proteins were used for immunoblotting, and for immunodetection of the AtJ8 protein, 30 μg protein was loaded whereas for immunodetection of AtJ11 and AtJ20 proteins, 100 μg protein was loaded. WT, wild-type.

In order to examine the localization of the three small DnaJ proteins, an antiserum for each protein was raised in rabbits using specific synthetic peptides. Despite purification of the antisera, we did not get good reactions using leaf total protein extracts (data not shown). However, as shown in Figure 2, the protein extracts from intact chloroplasts gave a specific band in WT around 17 kD, 15 kD, and 20 kD when the AtJ8, AtJ11 and AtJ20 antisera, respectively, were used, and importantly, the specific band was missing from the respective DnaJ mutant. This indicates that chloroplasts are at least one of the compartments containing these small DnaJ proteins in *Arabidopsis*. It should be noted that the size of each DnaJ protein in chloroplasts is somewhat lower than the predicted molecular mass (18.3-, 17.8- and 23.4-kD for AtJ8, AtJ11 and AtJ20, respectively). This is apparently due to the processing of the preprotein after import to chloroplast. In fact, Orme et al. reported that AtJ11 is located in chloroplast stroma and the mature protein has a molecular mass of 14.3 kD [7].

### Capacity of CO_2 _assimilation

**Figure 3:**
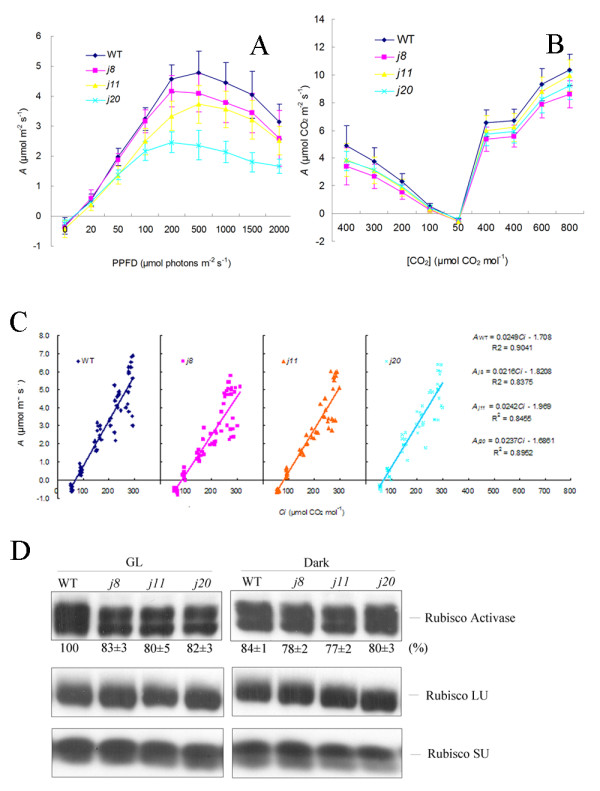
**Capacity of CO_2 _assimilation in DnaJ mutants and WT**. A, Light response curves; B, CO_2 _response curves; C, *A*-*Ci *curves which based on intracellular CO_2 _concentration less than 300 μmol mol^-1^; D, Immunoblot analysis of Rubisco Activase, Rubisco large subunit (Rubisco LU) and small subunit (Rubisco SU) in leaves collected from growth light conditions and from darkness. Total proteins were isolated from leaves after 6 h illumination under growth light and in the end of the diurnal dark period. 10 μg of leaf total proteins was loaded. Protein quantification (indicated below the blots as a percentage of protein from that present in WT in the light) is based on three independent immunoblot experiments (mean ± SD). *A*, CO_2 _assimilation; *Ci*, intracellular CO_2 _consentration; PPFD, photosynthetic photon flux density. WT, wild-type; GL, growth light (120 μmol photons m^-2 ^s^-1^).

To analyse whether the DnaJ proteins are involved in acquiring the maximal CO_2 _fixation capacity, we measured both the light response and CO_2 _response curves of the DnaJ mutants and WT. The light response curves showed the maximum CO_2 _assimilation rate at 500 μmol photons m^-2 ^s^-1 ^which then decreased with increasing photosynthetic photon flux density (PPFD) in both WT and the DnaJ mutants (Figure 3A). Compared to WT, the DnaJ mutants possessed lower CO_2 _assimilation, especially the *j20 *mutant. Relatively, the assimilation of *j8 *was only slightly lower as compared to WT, showing that the AtJ8 protein is less related to the light-dependent regulation of CO_2 _fixation. Nonetheless, the CO_2 _response curves revealed lower CO_2 _assimilation in *j8 *as compared to that in WT (Figure 3B). The *A-Ci *curves based on intracellular CO_2 _concentration less than 300 μmol mol^-1 ^demonstrated a lower Rubisco activity in all three DnaJ mutants as deduced from lower slope values of the curves as compared to WT, especially for *j8 *(Figure 3C). Although the amount of the Rubisco protein (large subunit and small subunit) did not obviously differ between WT and the DnaJ mutants, an immunoblot analysis of Rubisco Activase showed reduced amounts of this enzyme under light conditions in the DnaJ mutants as compared to WT (Figure 3D).

### Stabilization of PSII dimers and supercomplexes under high light illumination

**Figure 4:**
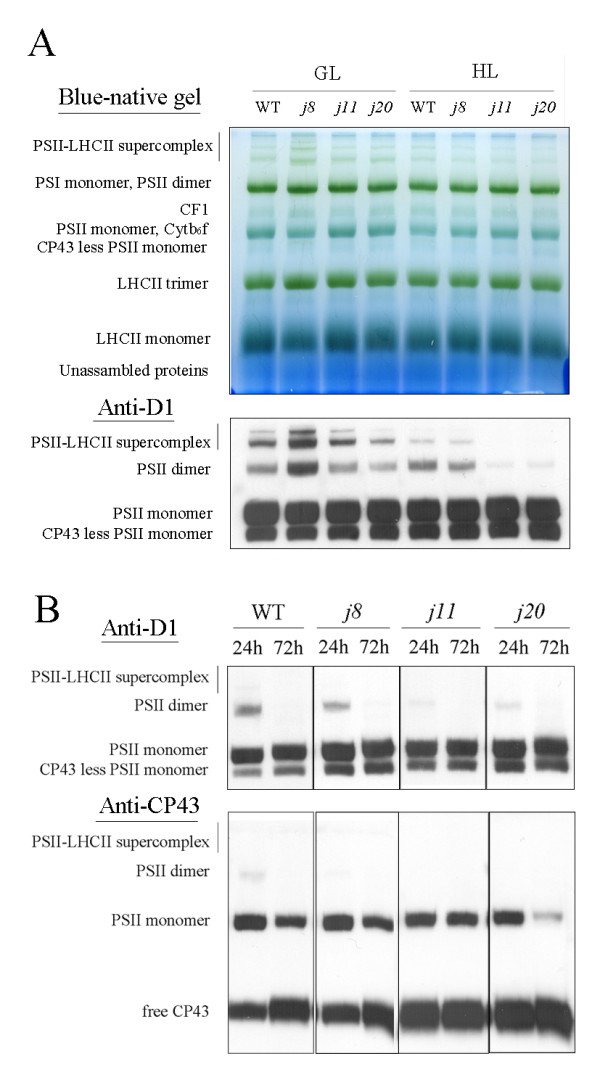
**BN-PAGE analysis of thylakoid protein complexes from WT and the DnaJ mutants**. Thylakoids corresponding 4 μg Chl were loaded in each lane. A, A BN gel of thylakoid protein complexes from plants exposed to growth light conditions for 6 h and from plants exposed to high light for 6 h. Top panel, BN gel directly after electrophoresis; lower panel, BN gel immunoblotted with D1 antibody. B, Immunoblots of the BN gels prepared from plants after a long-term high light (1000 μmol photons m^-2 ^s^-1^) exposure. Thylakoid membrane protein complexes of WT and the DnaJ mutants were subjected to Blue-native gel electrophoresis following immunoblotting with D1 (top panel) and CP43 (lower panel) antibodies. GL, 120 μmol photons m^-2 ^s^-1 ^growth light; HL, 1000 μmol photons m^-2 ^s^-1 ^high light.

Since the absence of one of the DnaJ proteins, AtJ8, AtJ11 or AtJ20, pronouncedly affected the photosynthetic capacity of respective mutants, we next investigated whether the DnaJ proteins are involved in regulation of the stability of the photosynthetic pigment protein complexes in the thylakoid membrane. Based on Blue-native gel electrophoretic (BN-PAGE) separation of thylakoid protein complexes (Figure 4A), the amount of PSII-LHCII supercomplexes was less in the DnaJ mutants than in WT after 6 h HL illumination (1000 μmol photons m^-2 ^s^-1^). Immunoblotting of the BN-gels with D1 antibody more clearly showed the decrease of PSII supercomplexes in the mutants after the HL treatment. Moreover, the amount of PSII dimers also significantly decreased in the DnaJ mutants upon the HL treatment, especially in *j11 *and *j20 *(Figure 4A). To get more insights into the function of the three DnaJ proteins in the maintenance of the PSII oligomers, a long-term treatment under HL was employed. As shown in Figure 4B, the PSII supercomplexes completely disappeared both from WT and the DnaJ mutants whereas the PSII dimers were much more stable in WT than in the DnaJ mutants in the course of the long-term HL treatment. As compared to WT, the DnaJ mutants *j11 *and *j20 *showed a total disappearance of PSII dimers already during 24 h of HL treatment (Figure 4B), and clearly more of CP43 proteins had released from PSII complexes at this time point as compared to WT or the *j8 *mutant. As the total amounts of the D1, D2, CP43, CF1 and NDH-H proteins were similar in WT and the three mutants even after the HL treatment (deduced from PAGE and immunoblotting - see Additional file 2), it can be concluded that the three DnaJ proteins do not participate in the biosynthesis of individual PSII core proteins, but only provide stability for the PSII protein complexes.

### Energy distribution between PSI and PSII and phosphorylation of the PSII-LHCII proteins

**Figure 5:**
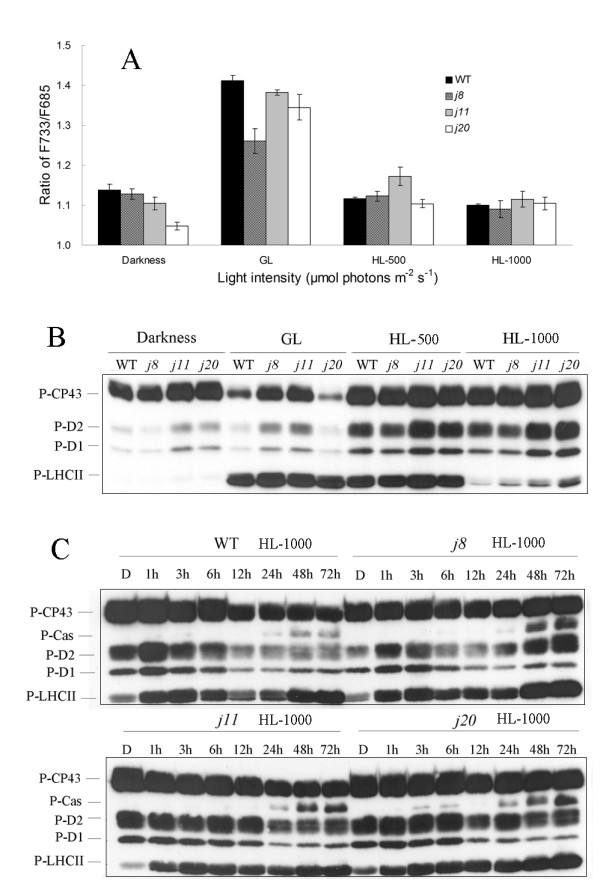
**The 77 K fluorescence emission ratio F733/F685 and the thylakoid protein phosphorylation in WT and the DnaJ mutants**. A, F733/F685 ratio in WT and the DnaJ mutants after 6 h treatment of plants under different light conditions. The values are means ± SD (n = 9~12) of three independent experiments with 3 to 4 replicates. B, Phosphorylation levels of thylakoid proteins after similar light treatments of plants as in A. C, Changes in thylakoid protein phosphorylation during a long-term high light (1000 μmol photons m^-2 ^s^-1^) treatment. Thylakoid membranes were isolated from leaves after treatment of plants in darkness and after illumination at growth light and high light conditions for time periods indicated. 1.0 μg of chlorophyll was loaded to the wells for immunoblotting with p-thr antibody. WT, wild-type; D, darkness; GL, 120 μmol photons m^-2 ^s^-1 ^growth light; HL-500, 500 μmol photons m^-2 ^s^-1^high light; HL-1000, 1000 μmol photons m^-2 ^s^-1 ^high light.

The 77 K chlorophyll fluorescence emission ratio F733/F685 was recorded as an indication of energy distribution between the PSI and PSII complexes (Figure 5A). The ratio of F733/F685 was slightly lower in the DnaJ mutants than in WT both when measured from dark acclimated and from GL acclimated plants. After HL illumination no clear differences in F733/F685 ratio were found between the WT and mutants with one exception, the ratio was higher in *j11 *as compared to that in WT after 500 μmol photons m^-2 ^s^-1 ^HL illumination (Figure 5A). To evaluate whether the phosphorylation of PSII proteins is related to redistribution of energy in plants lacking the DnaJ proteins, the phosphorylation levels of the major PSII phosphoproteins D1, D2, CP43 and LHCII were determined by immunoblotting with the P-Thr antibody. As can be seen in Figure 5B, only extremely weak phosphorylation of LHCII (P-LHCII) was detected in darkness and P-LHCII strongly accumulated in light conditions. Higher intensity light (1000 μmol photons m^-2 ^s^-1^) decreased the level of P-LHCII but did this less efficiently in the DnaJ mutants than in WT (Figure 5B). Interestingly, LHCII was phosphorylated to the same level in all strains under GL and moderate HL (500 μmol photons m^-2 ^s^-1^), despite clear differences in the 77 K fluorescence ratio under these two light conditions.

As to PSII core protein phosphorylation, under GL conditions the *j8 *and *j11 *mutants exhibited more P-CP43, P-D1 and P-D2 proteins as compared to WT while the *j20 *had less (Figure 5B). Under HL conditions (both 500 and 1000 μmol photons m^-2 ^s^-1^) the *j11 *and *j20 *mutants had a clearly higher level of PSII core protein phosphorylation. A long-term HL illumination (1000 μmol photons m^-2 ^s^-1^) experiment showed that fluctuations in phosphorylation of both the PSII core and LHCII proteins were characteristic for WT during acclimation to this HL condition. The *j8 *mutant showed similar fluctuations, though not as drastic as in WT (Figure 5C). The *j11 *and *j20 *mutants, however, differed from the WT and *j8*, showing clearly delayed and less obvious drop in the phosphorylation level of both the PSII core and LHCII proteins, which in WT and *j8 *occurred after 6 h illumination at HL whereas in *j11 *and *j20*, a less distinctive drop in phosphorylation was recorded after 12 - 24 h illumination at HL. Moreover in all DnaJ mutants, *j8*, *j11 *and *j20*, long HL illumination resulted in more drastic phosphorylation of the Cas protein (Figure 5C), a typical stress response of plants [17].

### Gene expression profiles

**Figure 6:**
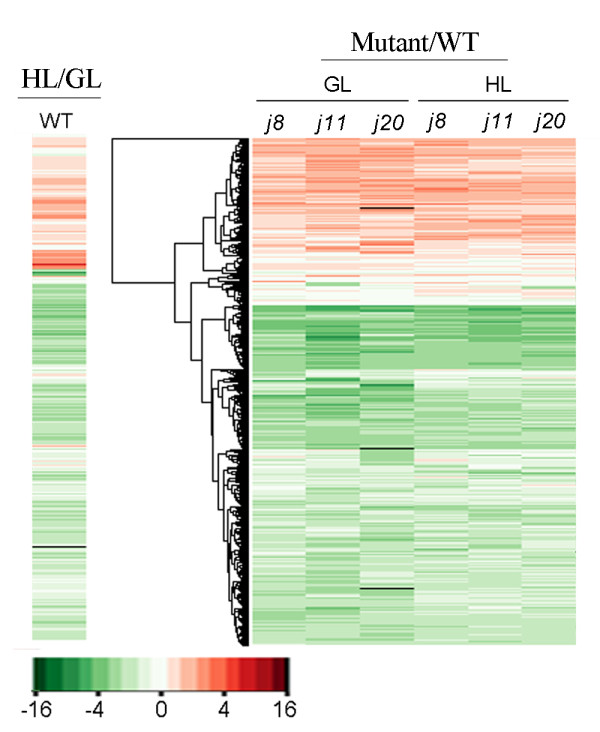
**Gene expression-profilings of the DnaJ mutants with comparison to WT**. Genes whose expression showed more than a two-fold change (up- or down-regulated) with the p-value less than 0.05 and the B-value more than 2.0 were selected for making the heatmaps using the R program and Bioconductor packages. The values are averages from three independent biological replicates starting from the growth of a new set of plants. The heatmap marked by WT shows the changes of gene expression in WT after 6 h illumination at 1000 μmol photons m^-2 ^s^-1 ^against 6 h illumination at 120 μmol photons m^-2 ^s^-1^. The heatmaps marked by the names of the DnaJ mutant show the changes of gene expression in each mutant against WT under both GL and HL conditions after 6 h illumination. WT, wild-type; GL, 120 μmol photons m^-2 ^s^-1 ^growth light; HL, 1000 μmol photons m^-2 ^s^-1 ^high light. (red, upregulated; green, downregulated; black, missing value).

**Figure 7:**
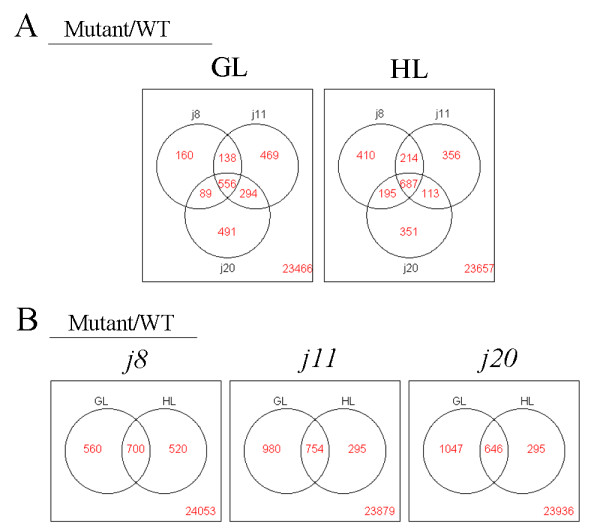
**Venn diagrams of genes impacted by a HL treatment and by a DnaJ protein knockout**. A, More than half of the genes changing expression are coregulated in the three DnaJ mutants and the mutants share 556 and 687 coregulated genes under GL and HL conditions, respectively. B, Coregulation analysis of gene expression between GL and HL conditions for each DnaJ mutant. WT, wild-type; GL, 120 μmol photons m^-2 ^s^-1 ^growth light; HL, 1000 μmol photons m^-2 ^s^-1 ^high light.

Based on somewhat similar effects on photosynthetic parameters of the knockout of any of the three small chloroplast targeted DnaJ proteins, it was of interest to analyse the gene expression profiles of these mutants. The expression of about 1,200 genes showed more than two-fold changes in WT by HL treatment, and among those genes one third were upregulated (Figure 6, Additional file 3). It was interesting to note that the gene expression profiles of the mutants showed similarities under both GL and HL conditions to the HL-treated WT, although the expression levels somewhat varied in each mutant (Figure 6). More than half of genes changing expression were found to be coregulated between the DnaJ mutants, and all three mutants shared 556 and 687 coregulated genes under GL and HL, respectively, indicating their very similar response between the DnaJ mutants (Figure 7A). In each mutant, the expression of roughly 700 genes had changed independently of the growth light condition (Figure 7B). It is also worth noting that the *j11 *and *j20 *mutants showed more divergent gene expression (920 and 1047 genes, respectively) at growth light from that in WT as compared to *j8 *(560 genes) whereas after HL treatment the reverse situation was recorded (Figure 7B). However, although the three DnaJ proteins are localized in the chloroplasts, most of the genes related to thylakoid membranes were not affected by lacking of one of the small DnaJ proteins (Additional file 4).

More interestingly, the DnaJ mutants showed stress-related regulation of several genes even at GL conditions. Expression of a number of genes related to transcription, translation and cellular signaling and to enzymes participating in the control of reactive oxygen species (ROS) and in redox regulation resembled that observed in WT upon transfer to HL (Additional file 4). Nevertheless, the DnaJ mutants also showed unique gene expression patterns from those induced in WT by HL treatment, including upregulation of several distinct genes encoding transcription factors, heat shock proteins, DnaJ proteins as well as antioxidant and redox proteins, among others (Additional file 4). Additionally, by using the MapMan tool, it was found that changes in expression of several genes related to distinct regulation pathways were quite similar in the DnaJ mutants at GL conditions to those recorded in the HL-treated WT (Additional file 5). Several clustered genes related to different functions, including hormone metabolism, stress response, redox regulation, transcriptional regulation, and protein degradation, were visualized and the results show that almost the same numbers of genes were regulated by HL in WT or by the lack of one DnaJ protein, AtJ8, AtJ11 or AtJ20 at GL conditions (Additional file 6). Particularly, the genes related to ubiquitin and ubiquitin E3 presented a high correlation between the HL stress response in WT and the DnaJ protein knockout (Additional file 6).

### Oxidative stress tolerance in the DnaJ mutants

**Figure 8:**
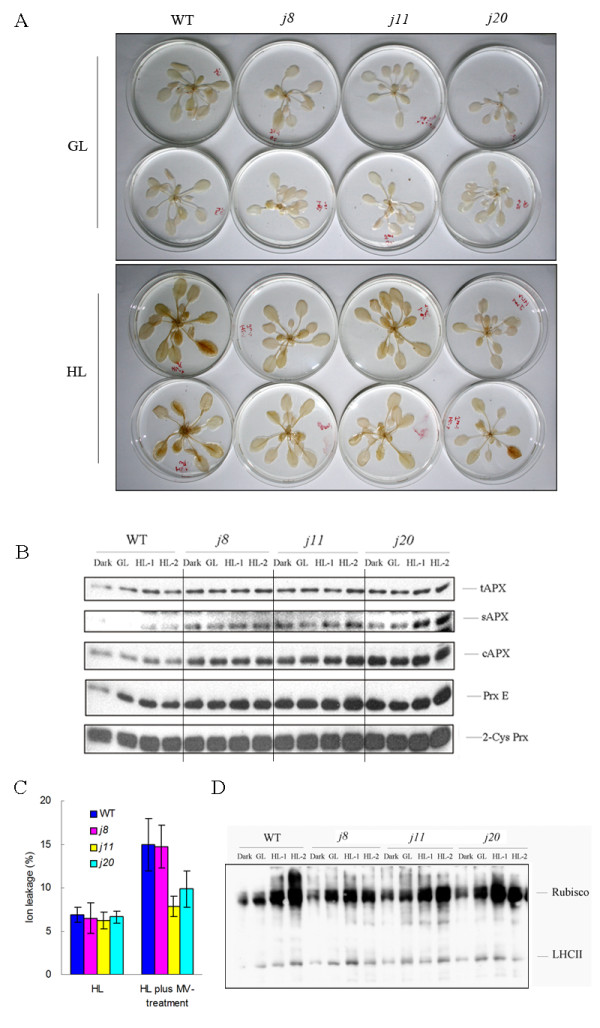
**Production of ROS and the stress tolerance of WT and the DnaJ mutant *j8*, *j11 *and *j20***. A, Histochemical detection of H_2_O_2 _in the leaves with DAB staining after 6 h incubation of leaves under GL (120 μmol photons m^-2 ^s^-1^) and HL (1000 μmol photons m^-2 ^s^-1^). B, Immunoblots depicting the levels of H_2_O_2_-detoxifying enzymes in WT and the DnaJ mutant leaves after 6 h incubation of plants under different light conditions. 10 μg of the leaf total proteins loaded. C, Ion leakage induced by 6 h HL (1000 μmol photons m^-2 ^s^-1^) illumination of leaves in the presence and absence of Methyl viologen (MV), the values are means ± SD (n = 8) of two independent experiments with 4 replicates. D, OxyBlot of leaf total proteins (10 μg proteins loaded) after treatment of plants at different light intensities. GL, 120 μmol photons m^-2 ^s^-1 ^growth light; HL-1, 500 μmol photons m^-2 ^s^-1 ^high light; HL-2, 1000 μmol photons m^-2 ^s^-1 ^high light. WT, wild-type.

Based on the cues from microarray results, we next tested some oxidative stress responses of the DnaJ mutants. At first, the H_2_O_2 _levels in the leaves of the DnaJ mutants and WT were detected using DAB (diaminobenzidine) as a substrate. Notably, the staining intensity and accordingly the level of H_2_O_2 _in the DnaJ mutants was lower as compared to WT, especially in plants illuminated under HL for 6 h (Figure 8A). Since ascorbate peroxidases (APXs) and chloroplast peroxiredoxins (PRXs) associated with the water-water cycle, are generally known protectants of chloroplasts against oxidative damage, the contents of these H_2_O_2_-detoxifying enzymes were evaluated by immunoblotting. As shown in Figure 8B, the amounts of all these enzymes, including tAPX (thylakoid APX), sAPX (stroma APX), cAPX (cytoplasmic APX), and two PRXs, PrxE (peroxiredoxin E) and 2-Cys Prx (2-cysteine peroxiredoxin), were pronouncedly higher in the three DnaJ mutants as compared to WT no matter whether the plants were subjected to darkness, GL or HL conditions before measurements. Nevertheless, higher amounts of these enzymes were present in the light conditions, especially in HL. These results suggest that the higher amounts of H_2_O_2_-detoxifying enzymes contributed to the lower H_2_O_2 _levels in the mutants.

To investigate the tolerance of the DnaJ mutants to oxidative stress, 50 μM methyl viologen (MV) was supplied to plants followed by illumination at 1000 μmol photons m^-2 ^s^-1 ^HL for 6 h, and the cellular ion leakage of whole plant rosettes was determined. Plants untreated with MV showed no differences in ion leakage between the DnaJ mutants and WT. In WT plants the MV treatment strongly enhanced ion leakage levels, whereas in the *j11 *and *j20 *mutant plants the ion leakage was only slightly increased, indicating that the mutants had better resistance to MV-induced oxidative stress (Figure 8C, Additional file 1). Although *j8 *exhibited similar levels of ion leakage as WT in MV treated plants, the oxidation level of leaf total proteins isolated from the mutant was less severe than that in WT after the HL treatment (Figure 8D). In general, the DnaJ mutants showed less oxidation of leaf total proteins, particularly the Rubisco protein, as a response to environmental light intensity changes as compared to WT (Figure 8D).

## Discussion

The DnaJ proteins assist the Hsp70 chaperone proteins, participating in protein folding, unfolding, and assembly processes [1,2]. Such functions, based mainly on biochemical experiments, are still unproven for the chloroplast DnaJ proteins, and their physiological roles remain largely unknown. Here we particularly focused our study on the physiological role of the three small chloroplast-targeted DnaJ proteins, AtJ8, AtJ11 and AtJ20 which according to our database searches, only contain the J-domain (data not shown) and thus possibly have divergent functions from ordinary DnaJ proteins. Although we did not obtain comprehensive information about the localization of the three small DnaJ proteins in different cell compartments, the immunoblotting experiments with protein extracts from isolated chloroplasts clearly showed that the three DnaJ proteins are targeted to chloroplasts in *Arabidopsis *(Figure 2).

### Small chloroplast-targeted DnaJ proteins participate in regulation of CO_2 _fixation and in stabilization of PSII supercomplexes and dimers

Due to chloroplast location of the three small DnaJ proteins, we applied simultaneous measurements of the responses of leaf gas exchange to light and CO_2 _concentration [18,19], which provided first evidence of limitation of the *in vivo *photosynthesis in all the three DnaJ mutants. Both the lower slopes of light response curves and lower CO_2 _fixation at light saturation in the mutants (particularly in *j11 *and *j20*) (Figure 3) imply limitations in electron transport required for RuBP regeneration, Rubisco activity, or metabolism of triose phosphates [18,19]. The *A*-*Ci *curves confirmed that particularly the activity of Rubisco is compromised in the DnaJ mutants, especially in *j8*. Considering the general function of the DnaJ proteins as chaperone proteins [1], the reduced amount of Rubisco Activase in the mutants reported here, suggests that the three small chloroplast DnaJ proteins are involved in the folding, unfolding, or assembly processes of this enzyme and thus participate in regulation of Rubisco activity [20]. It should be noted that the functional mechanisms between the DnaJ proteins AtJ8, AtJ11 and AtJ20 in regulation of CO_2 _fixation might be somewhat different because the *j8 *mutant differed in the response to ambient CO_2 _concentration while the *j11 *and *j20 *mutants showed lower response to light intensity, which suggests the involvement of photosynthetic light reactions in limitation of CO_2 _fixation. Somewhat lower photochemical efficiency of PSII, the Fv/Fm ratio, particularly in *j11 *and *j20 *mutants after the HL treatment refers to malfunction of PSII. Moreover, the HL treatments of WT and the DnaJ mutant plants suggested the involvement of AtJ11 and AtJ20 in stabilization of PSII supercomplexes and dimers.

The deduction that these small chloroplast-targeted DnaJ proteins are related to regulation of CO_2 _assimilation is also supported by the data from DNA microarray studies. Several genes related to the Calvin-Benson cycle showed big changes in expression in the DnaJ mutants as compared to WT (Additional file 4). Particularly the expression of Ribose 5-phosphate isomerase was significantly downregulated in the mutants under both GL and HL conditions, implying this downregulation as one of the reasons for, or a consequence of, the lower CO_2 _assimilation in the mutants. On the contrary, the expression of most genes related to thylakoid membrane proteins was not changed in the mutants, neither under GL nor under HL conditions as compared to WT (Additional file 4). The expression of only the FZL-like protein gene was significantly downregulated in *j11 *and *j20 *under GL and in all the three mutants under HL (Additional file 4), implying that the FZL-like protein might function in co-operation with the DnaJ proteins and control the aggregation/disaggregation of PSII complexes since the FZL-like protein regulates the organization of the thylakoid network in chloroplasts [21].

### Knocking out one of the small chloroplast-targeted DnaJ proteins modifies the capacity for dynamic regulation of chloroplast redox reactions

Energy distribution between PSII and PSI is regulated by phosphorylation of the PSII-LHCII complexes [22], which in turn is strongly dependent on the redox state of electron transfer components in the thylakoid membrane as well as in the soluble stroma [23]. Although the differences in PSII-LHCII protein phosphorylation between both the three DnaJ mutants and the different light intensities did not allow to draw any strict conclusions about energy distribution between PSII and PSI, it was clearly evident that more phosphorylated thylakoid proteins accumulated in the DnaJ mutants, particularly *j11 *and *j20 *during all different short-term illumination conditions (Figure 5B and 5C). This prompted us to analyse the thylakoid phosphoprotein profiles in the course of long-term HL illumination of both the WT and DnaJ mutant plants. Indeed, the changes in the phosphorylation pattern of thylakoid proteins revealed the capacity of the electron transfer chain to acclimate to changes in light conditions. WT clearly showed a strong phosphorylation of PSII core proteins immediately after exposure to HL, which also reflects a high reduction state of the plastoquinone (PQ) pool [23]. Nevertheless, the dynamics of thylakoid functions allowed re-oxidation of the PQ pool in the course of HL illumination and already after 6 h this was reflected in lower phosphorylation level of the PSII core proteins D1, D2 and CP43. *j8 *mutant also had such a capacity to respond to the HL treatment. The *j11 *and *j20 *mutant plants showed clearly less capacity for acclimation of the thylakoid redox reactions to prolonged exposure to HL and kept the PSII core proteins strongly phosphorylated during the entire HL treatment, implying highly reduced PQ pool. Similarly, a more severe stress response of the mutants as compared to WT after prolonged HL treatment can be deduced from the strong phosphorylation of the Cas protein, reflecting highly reduced electron transfer chain in all mutants [17]. Cas protein is a calcium-sensing receptor that was found to be located in the stroma thylakoids of chloroplasts and functions in stress responses and signaling pathways [17].

### Knocking out any one of the small chloroplast-targeted DnaJ protein triggers a global stress response

As discussed above, the knocking out of any one of the three small chloroplast-targeted DnaJ proteins AtJ8, AtJ11 or AtJ20 causes many events in *Arabidopsis*, which are reminiscent of generally known stress responses in plants. The most typical response is the increased tolerance of the mutant plants to oxidative stress induced by HL and MV (Figure 8). It was recently reported that the DnaJ family proteins participate in H_2_O_2_-induced gene expression matrix in higher plants as well as in yeast and cyanobacteria [24]. The results reported here likewise show that the DnaJ proteins are involved in ROS-induced stress responses in *Arabidopsis*. The gene expression profiles of the DnaJ mutants, even under GL conditions, are quite similar to those induced upon ROS-producing HL treatment in WT (Figure 6). Moreover, the transcripts of some specific antioxidant genes, like *apx6*, *cat1*, *CSD3*, *gpx5*, *glutaredoxin-like*, were found to be specifically upregulated only in the three small chloroplast-targeted DnaJ knockout mutants (Additional file 3 and 4). Such upregulation of ROS scavenging and antioxidant systems coincided with lower H_2_O_2 _content in the DnaJ mutants than in WT, both at GL and HL conditions (Figure 8). Many genes encoding intracellular redox regulators such as thioredoxins and glutaredoxins with strong impact on stress tolerance [25] were upregulated together with several receptor kinases and G-proteins in the DnaJ mutants (Additional file 5), implying redox regulation in the stress tolerance of the DnaJ mutants. Moreover, the expression of a number of genes encoding conventional transcription factors and many novel ones containing zinc-finger, MYB, NAC and AP2 domains, which are tightly correlated with stress responses [26], showed significant modifications in the DnaJ mutants even under GL conditions (Additional file 3 and 4). Many of their target genes are likely involved in protein modification and degradation processes, as can be deduced from the MapMan analysis (Additional file 5). These results strongly indicate that a global stress response has been triggered in the three DnaJ mutants even in the absence of external stress.

The comparison of the gene expression profiles of our DnaJ mutants with those of other mutants deposited in the public microarray database further supported the idea that the tolerance of the DnaJ mutants to oxidative stress induced by MV is due to the trigger of a global stress response in these mutants (Additional file 6 and 7). The expression of 70 genes co-downregulated in the three DnaJ mutants is quite similar to that observed for oxidative stress related mutants of *Arabidopsis*, *oxt6 *and *over*-tAPX mutants. *Oxt6 *displays more tolerance to oxidative stress [25] whereas *over*-tAPX is a transgenic line which overexpresses the thylakoid-bound ascorbate peroxidase [27]. On the contrary, completely opposite regulation of the 70 genes was displayed by HSP90(RNAi-A3), a HSP90-reduced line of *Arabidopsis *showing enhanced sensitivity to high temperature and to pathogen attack [28], and by the CSN mutants [29]. The most studied CSN function is the regulation of protein degradation and beyond this, the CSN also acts as a transcriptional regulator [30].

## Conclusion

The functions of the three small chloroplast-targeted DnaJ proteins AtJ8, AtJ11 and AtJ20 seem to be, at least partially, redundant in *Arabidopsis*, yet it is evident that also specific functions exist for each protein. Knockout of the AtJ8 protein revealed less drastic influence on photosynthetic parameters than the knockout of either the AtJ11 or the AtJ20 protein. Because of the small sizes and the lack of client protein-interaction domains, the multiple, yet subtle, effects on photosynthesis performance induced by the knockout of any one of the small DnaJ proteins, AtJ8, AtJ11 or AtJ20, makes it hard to distinguish the individual roles of these DnaJ proteins in co-chaperone/chaperone cohort. Nevertheless, in general, these small chloroplast DnaJ proteins participate in optimization of CO_2 _fixation, in stabilization of PSII complexes and balancing the electron transfer reactions. It is conceivable that the tolerance of the DnaJ protein knockout plants to oxidative stress results from an unbalance of the redox reactions in chloroplasts, thereby modifying the chloroplast retrograde signaling mechanisms and inducing the up- or down-regulation of stress responsive genes in the nucleus [16,31].

As a whole, it can be concluded that both specific- and cross-talk functions exist between the three small chloroplast-targeted DnaJ proteins, and the tolerance of the DnaJ protein knockout plants to oxidative stress occurs at the expense of the flexibility of photosynthetic reactions. Further studies with double and triple mutants are expected to provide stronger phenotypes and also to give deeper insights into the functions of the AtJ8, AtJ11 and AtJ20 proteins in chloroplasts.

## Methods

### Plant materials, growth conditions and high light treatments

*Arabidopsis thaliana *ecotype Columbia (Col-0) wild-type (WT) and homozygous T-DNA insertion mutants for chloroplast-targeted DnaJ proteins, At1 g80920, At4 g36040, and At4 g13830, (*j8*, *j11 *and *j20*, respectively) were used in the experiments. The mutant lines salk_024617, salk_015630, and salk_134365 for AtJ8, AtJ11 and AtJ20, respectively, were selected to screen the homozygotes by standard PCR protocols recommended by SIGnAL (SALK, USA). Other mutant lines for the AtJ11 and AtJ20 proteins were salk_052270 and salk_125167, respectively, the results of which are presented in the Supplemental materials. Plants were grown under standard control conditions as described previously [32]. High light treatments (500 and 1000 μmol photons m^-2 ^s^-1^) were given using the same light source and were started immediately after the diurnal dark period. Mature rosette leaves from 4 to 6 weeks old plants were used for experiments.

### Chlorophyll and protein determinations

Chlorophyll was determined according to Inskeep & Bloom [33] and Porra *et al*. [34] from leaf discs and isolated thylakoids, respectively. The protein content was determined using a *DC *(detergent-compatible) protein assay kit (Bio-Rad, Hercules, CA).

### Measurements of PSII photochemical efficiency, 77 K chlorophyll fluorescence spectra, and CO_2 _assimilation

PSII photochemical efficiency was determined as a ratio of variable fluorescence (Fv) to maximal fluorescence (Fm) measured from intact leaves with a Hansatech Plant Efficiency Analyser (Hansatech, King's Lynn, UK) after a dark incubation of 30 min. 77 K chlorophyll fluorescence emission spectra were measured as described by Soitamo *et al*. [26]. CO_2 _assimilation was determined using an open-gas portable photosynthesis system (LI-6400, LI-COR, Lincoln, Nebraska, USA) with artificial blue-red light emitting diodes (LED) source. The response of CO_2 _assimilation to photosynthetic photon flux density (PPFD) was carried out by varying the PPFD from 2000 μmol m^-2 ^s^-1 ^to zero in the presence of 400 μmol mol^-1 ^of CO_2_. The response of CO_2 _assimilation to CO_2 _concentration was determined at 1000 μmol m^-2 ^s^-1 ^of PPFD. 10 to 15 plants were measured for both the light response curves and the CO_2 _response curves based on three independent biological repeats.

### Preparation of antibodies against the three DnaJ proteins

The antibodies for AtJ8, AtJ11 and AtJ20 were made by Innovagen Company (Lund, Sweden). Two rabbits were immunized using a specific synthetic peptide for each protein: AtJ8 (111-134) (NH2-) CKNQMEGTEEFEPFDVYDEGLNGMN (-CONH2); AtJ11 (127-136) (NH2-) CSVYDRRMLRR (-CONH2); AtJ20 (145-158) (NH2-) CRQNRYDQEVVEEKS (-CONH2).

### Isolation of intact chloroplasts, thylakoid membranes and total leaf protein extracts

Intact chloroplasts were isolated from mature *Arabidopsis *leaves using a two-step Percoll gradient as described previously [17] and the thylakoid membranes as described by Suorsa *et al*. [35]. For the total leaf protein extraction, the leaf disks of equal size were cut from mature leaves and carefully homogenized in grinding buffer, composed of shock buffer (10 mM HEPES-KOH, pH 7.6, 5 mM sorbitol, 5 mM MgCl_2 _and 10 mM NaF) and solubilization buffer [36] (1:1, v:v).

### Blue-native-PAGE, SDS-PAGE and immunoblotting

Proteins were separated by SDS-PAGE using 15% (w/v) acrylamide gels with 6 M urea [36]. After electrophoresis, proteins were electroblotted to a polyvinylidene fluoride (PVDF) membrane (Millipore, Watford, Herts, UK), and subsequently blocked with 5% milk (for immunoblotting with AtJ8, AtJ11, AtJ20, D1, D2, CP43, CF1, NDH-H, Rubisco, and Rubisco Activase antibodies) or fatty acid free BSA (for immunobloting with Phosphothreonine (P-Thr) antibody and APXs and PRXs antibodies). P-Thr-specific antibody was purchased from New England Biolabs [37]. APXs and PRXs antibodies were obtained as described previously [32]. Other protein-specific antibodies were purchased from Research Genetics (D1 and D2), or were kindly provided by Dr Heather J. Kane (Rubisco and Rubisco Activase), Dr Roberto Barbato (CP43), Dr Torill Hundal (CF1), and Dr Gilles Peltier (NDH-H). Preparation of samples and detection of protein oxidation were performed according to the protocol of the OxyBlot protein oxidation detection kit (Intergen, Purchase, NY, USA). The protein amounts loaded in the gels were carefully controlled to fall into the linear range of the antibody response curves.

Blue-native-PAGE was performed as described previously by Rokka *et al*. [38]. After electrophoresis, the gels were photographed and then shortly destained by methanol. The destained gels were used for immunoblotting with D1 and CP43 antibodies, respectively.

### In vivo H_2_O_2 _detection and ion leakage measurement

Accumulation of H_2_O_2 _in leaves was detected using DAB (diaminobenzidine; Sigma-Aldrich, USA) as described in Kangasjärvi *et al*. [32]. The sensitivity of expanded 5-week old rosettes to Methyl viologen (MV)-induced photo-oxidative stress was determined by gently spraying the plants with 50 μM MV at the end of the dark period as described in Kangasjärvi *et al*. [32].

### Microarray analysis

Global changes in gene expression were explored with spotted *Arabidopsis *24 k oligonucleotide arrays (MWG Biotech; ArrayExpress database accession number A-ATMX-2, Ireland). Plant rosettes, 4-weeks old, of wild-type and the DnaJ mutants, *j8*, *j11 *and *j20*, were collected from GL and from HL conditions after illumination for 6 h following the diurnal dark period. Total RNA was isolated with TRIzol-reagent as described previously [16] and subsequently followed as described by Kangasjärvi *et al*. [32]. The arrays were scanned with an Agilent scanner, and the spot intensities were quantified with ScanArray Express Microarray Analysis system 2.0 (PerkinElmer Life Sciences, USA). The data from three biological replicates were analysed with the R program and Bioconductor packages. Average expression for each line and treatment was calculated and only the genes whose expression showed more than two-fold changes (upregulated or downregulated) with the p-value less than 0.05 and the B-value more than 2.0 were used for making the heatmaps and Venn diagrams. The B-value is the log-odds that the gene is differentially expressed [39]. The higher B-value means the higher probability of gene differential expression.

## Authors' contributions

K-M C carried out microarray experiments from plant material to data analysis; Fv/Fm and 77 K measurements, CO_2 _assimilation assay, Western blot analysis, Blue-native PAGE and writing the article. MH participated in technical support. WR carried out the tests of DnaJ antibodies. MS participated in manuscript revising. MP contributed to mutant screening. E-M A participated in planning the experiments, reading and revising the manuscript. All authors read and approved the final manuscript.

## Supplementary Material

Additional file 1 **Characterization of alterative mutant lines for AtJ11 and AtJ20**. Characteristics of the two alternative mutant lines found for the DnaJ proteins AtJ11 and AtJ20 (salk_052270 and salk_125167, respectively) were found to be similar with those described in the main text. A, Morphology of mutants; B, PSII photochemical efficiency of the DnaJ mutants, showing lower ratios of Fv/Fm in the mutants as compared to that of WT after 6 h high light treatment, the values are means ± SD (n = 10) of ten independent experiments; C, Ion leakage induced by 6 h HL (1000 μmol photons m^-2 ^s^-1^) illumination of leaves in the presence and absence of Methyl viologen (MV), the values are means ± SD (n = 8) of two independent experiments with 4 replicates. WT, wild-type; GL, growth light (120 μmol photons m^-2 ^s^-1^); HL, high light (1000 μmol photons m^-2 ^s^-1^). Click here for file

Additional file 2 **Immunoblot analysis of thylakoid proteins in WT and the DnaJ mutants**. Thylakoids were isolated after 6 h treatment of plants under different light conditions (darkness, growth light and high light of either 500 or 1000 μmol photons m^-2 ^s^-1^) and subjected to denaturaling gel electrophoresis. From 0.2 to 2.0 μg of chlorophyll were loaded in the wells depending on the linearity test with each antibody. WT, wild-type; GL, 120 μmol photons m^-2 ^s^-1 ^growth light; HL-1, 500 μmol photons m^-2 ^s^-1 ^high light; HL-2, 1000 μmol photons m^-2 ^s^-1 ^high light. Click here for file

Additional file 3 **Gene expression lists for WT and the DnaJ mutants**. For WT, 6 h high light illumination was compared to 6 h growth light illumination, and for the DnaJ mutants, 6 h illumination at growth light or at high light was compared to WT illuminated under similar conditions as the mutants. The genes whose expression changed in average more than 2.0 fold compared to GL WT samples (upregulated or downregulated) were selected using the R program and Bioconductor packages. Click here for file

Additional file 4 **Selected groups of genes, their expression changes and the p-values from the microarray experiment**. The column designated by WT shows the absolute ratios of gene expression in wild-type after 6 h HL treatment compared to 6 h GL treatment. The columns marked by DnaJ mutant names show the ratios of gene expression in each mutant against WT under both growth light and high light illumination for 6 h. C.fold, change folds of gene expression; p, the p-value of results of the students t-test for each comparison. WT, wild-type; GL, 120 μmol photons m^-2 ^s^-1 ^growth light; HL, 1000 μmol photons m^-2 ^s^-1 ^high light. Click here for file

Additional file 5 **Differential expression related to various regulation pathways in the DnaJ mutants and WT**. For WT, 6 h high light illumination was compared to 6 h growth light illumination, and for the DnaJ mutants, 6 h illumination at growth light was compared to WT illuminated under similar conditions as the mutants. For the analysis with MapMan, the genes whose expression changed in average more than 1.5 fold compared to GL WT samples (upregulated or downregulated) with p-value less than 0.05 were submitted to MapMan to determine the enrichment in a specific biological process. Click here for file

Additional file 6 **Number of differentially expressed genes related to specific regulation pathways and stress tolerance reactions in the DnaJ mutants and WT**. Genes whose expression showed more than-1.5 fold difference with the p-value less than 0.05 and the B-value more than 2.0 were selected using the R program. Visualization of clustered genes was done using the MapMan tool based on the Wilcoxon Rank Sum Test. The column designed by WT shows the number of genes differentially expressed in WT after 6 h 1000 μmol photons m^-2 ^s^-1 ^high light treatment related to that after 6 h treatment at 120 μmol photons m^-2 ^s^-1^. The columns marked by DnaJ mutant shows the number of genes differentially expressed in each mutant against the wild-type under growth light conditions after 6 h illumination. WT, wild-type; GL, 120 μmol photons m^-2 ^s^-1 ^growth light; HL, 1000 μmol photons m^-2 ^s^-1 ^high light. Click here for file

Additional file 7 **Comparison of gene expression profiles between the three DnaJ mutants and the mutants published in the public microarray database**. Total 70 co-downregulated genes with more than 4.2 fold expression changes in the mutants as compared to WT were selected by the R program with the p-value less than 0.01 and the B-value more than 4.0. The microarray data published in the Genevestigator Microarray Database https://www.genevestigator.com/gv/index.jsp was taken under investigation and the Genevestigator V3 program was used to get the comparison of gene expression. Mutants with closely similar expression as in the DnaJ mutants of the 70 genes included *oxt6*, an oxidative stress tolerant mutant of *Arabidopsis *and *over-tAPX*, a transgenic line that overexpresses the thylakoid-bound ascorbate peroxidase. On the contrary, HSP90(RNAi-A3), a HSP90-Reduced line of *Arabidopsis*, *cns3-1*, a mutant of Constitutive Photomorphogenic 9 (CSN) subunit 3 and *cns4-1*, a mutant of CSN subunit 4 showed an opposite expression profile of the 70 genes co-regulated in the three DnaJ mutants. Click here for file
