# Supplementary figures and images for: Small chloroplast-targeted DnaJ proteins are involved in optimization of photosynthetic reactions in Arabidopsis thaliana
Source: BMC Plant Biol. 2010 Mar 7;10:43. doi: 10.1186/1471-2229-10-43 (PMC2844072; doi:10.1186/1471-2229-10-43)

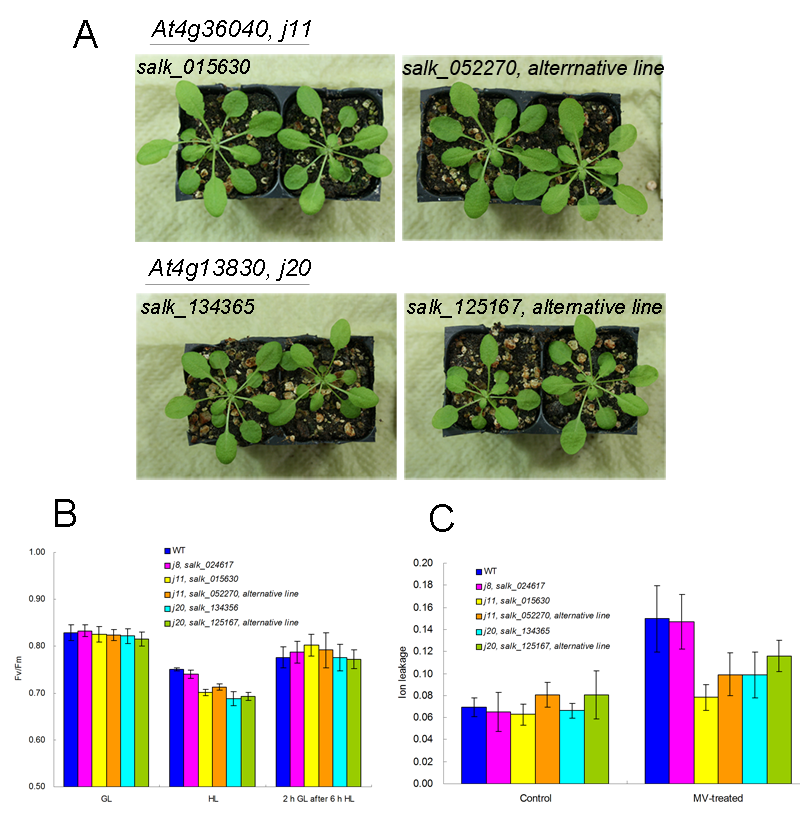

Supplement: Additional file 1 — Characterization of alterative mutant lines for AtJ11 and AtJ20. Characteristics of the two alternative mutant lines found for the DnaJ proteins AtJ11 and AtJ20 (salk_052270 and salk_125167, respectively) were found to be similar with those described in the main text. A, Morphology of mutants; B, PSII photochemical efficiency of the DnaJ mutants, showing lower ratios of Fv/Fm in the mutants as compared to that of WT after 6 h high light treatment, the values are means ± SD (n = 10) of ten independent experiments; C, Ion leakage induced by 6 h HL (1000 μmol photons m-2 s-1) illumination of leaves in the presence and absence of Methyl viologen (MV), the values are means ± SD (n = 8) of two independent experiments with 4 replicates. WT, wild-type; GL, growth light (120 μmol photons m-2 s-1); HL, high light (1000 μmol photons m-2 s-1). [file 1471-2229-10-43-S1.TIFF]

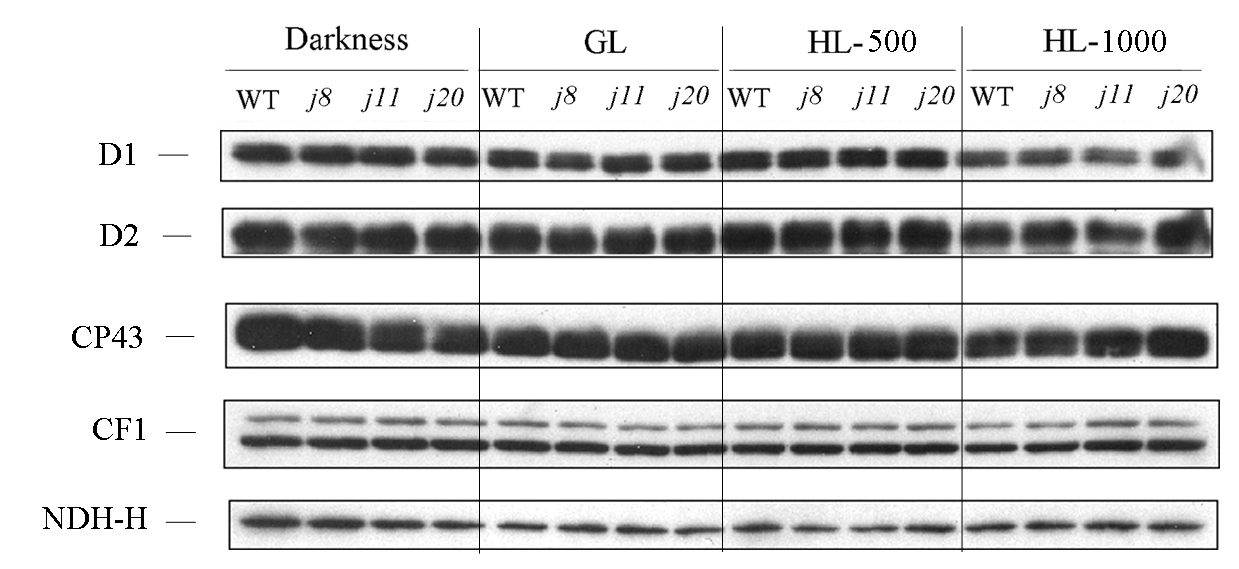

Supplement: Additional file 2 — Immunoblot analysis of thylakoid proteins in WT and the DnaJ mutants. Thylakoids were isolated after 6 h treatment of plants under different light conditions (darkness, growth light and high light of either 500 or 1000 μmol photons m-2 s-1) and subjected to denaturaling gel electrophoresis. From 0.2 to 2.0 μg of chlorophyll were loaded in the wells depending on the linearity test with each antibody. WT, wild-type; GL, 120 μmol photons m-2 s-1 growth light; HL-1, 500 μmol photons m-2 s-1 high light; HL-2, 1000 μmol photons m-2 s-1 high light. [file 1471-2229-10-43-S2.TIFF]

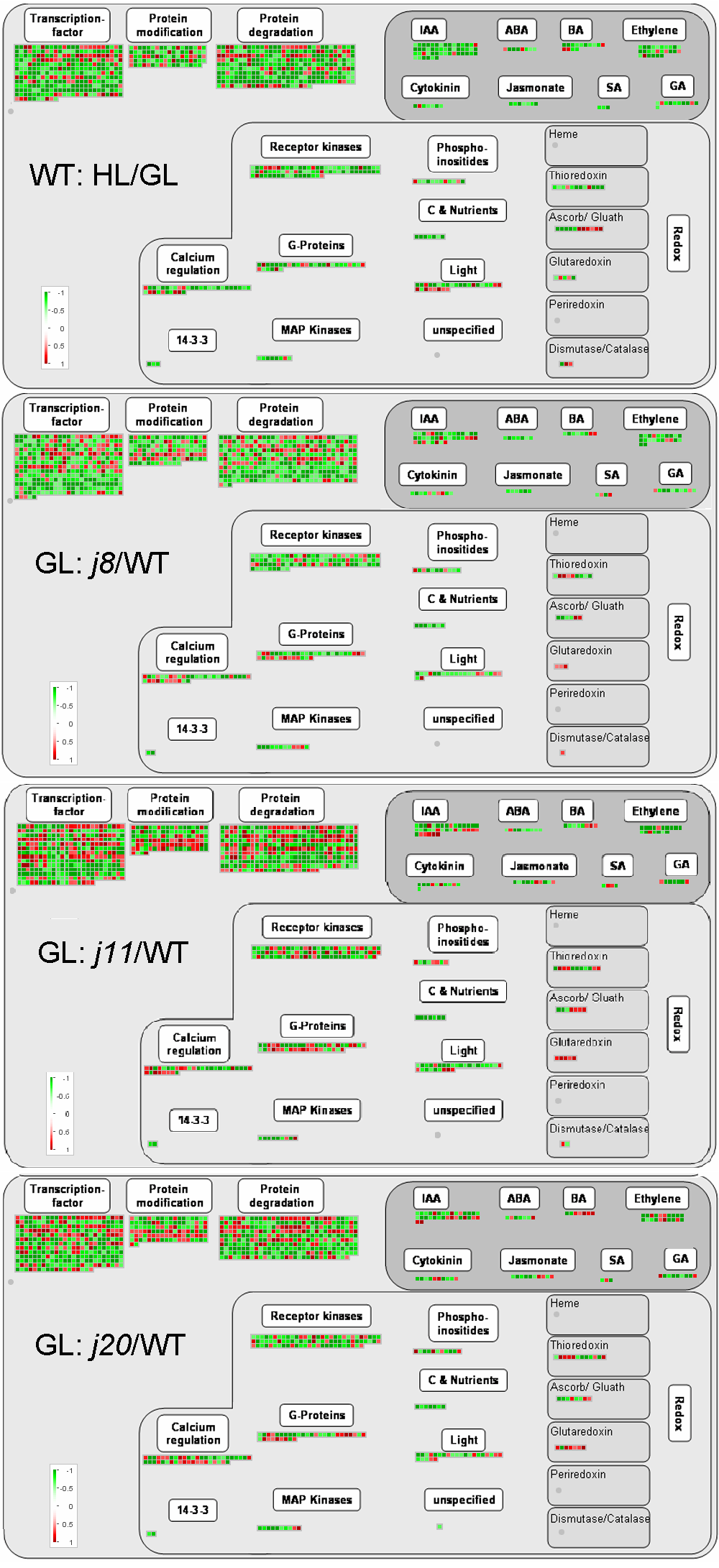

Supplement: Additional file 5 — Differential expression related to various regulation pathways in the DnaJ mutants and WT. For WT, 6 h high light illumination was compared to 6 h growth light illumination, and for the DnaJ mutants, 6 h illumination at growth light was compared to WT illuminated under similar conditions as the mutants. For the analysis with MapMan, the genes whose expression changed in average more than 1.5 fold compared to GL WT samples (upregulated or downregulated) with p-value less than 0.05 were submitted to MapMan to determine the enrichment in a specific biological process. [file 1471-2229-10-43-S5.TIFF]

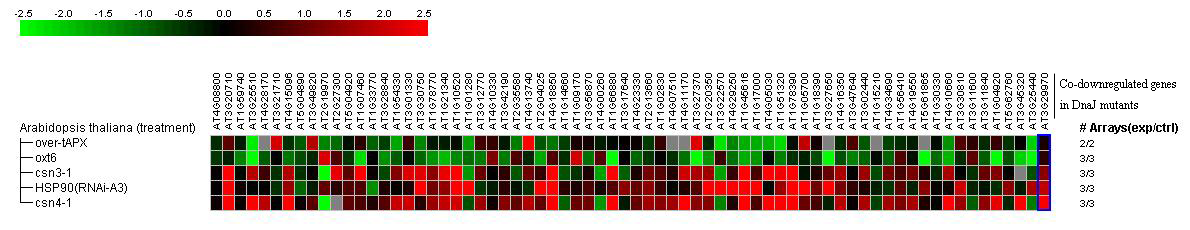

Supplement: Additional file 7 — Comparison of gene expression profiles between the three DnaJ mutants and the mutants published in the public microarray database. Total 70 co-downregulated genes with more than 4.2 fold expression changes in the mutants as compared to WT were selected by the R program with the p-value less than 0.01 and the B-value more than 4.0. The microarray data published in the Genevestigator Microarray Database https://www.genevestigator.com/gv/index.jsp was taken under investigation and the Genevestigator V3 program was used to get the comparison of gene expression. Mutants with closely similar expression as in the DnaJ mutants of the 70 genes included oxt6, an oxidative stress tolerant mutant of Arabidopsis and over-tAPX, a transgenic line that overexpresses the thylakoid-bound ascorbate peroxidase. On the contrary, HSP90(RNAi-A3), a HSP90-Reduced line of Arabidopsis, cns3-1, a mutant of Constitutive Photomorphogenic 9 (CSN) subunit 3 and cns4-1, a mutant of CSN subunit 4 showed an opposite expression profile of the 70 genes co-regulated in the three DnaJ mutants. [file 1471-2229-10-43-S7.TIFF]
